# Greater Interference From Multiple Exposures During Memory Retrieval Drives More Memorable and Forgettable Experiences

**DOI:** 10.1002/hipo.70057

**Published:** 2025-12-23

**Authors:** Fernanda Morales‐Calva, Aditi Velgekar, Stephanie L. Leal

**Affiliations:** ^1^ Department of Psychological Sciences Rice University Houston Texas USA; ^2^ Department of Integrative Biology & Physiology UCLA Los Angeles California USA

**Keywords:** episodic memory, hippocampus, long‐term memory, memorability, memory, perception

## Abstract

Our everyday experiences share many overlapping features, as we often engage in repeated activities and routines. This leads to interference across our experiences, making it difficult to remember specific, unique events. Hippocampal pattern separation enables the discrimination of highly similar experiences to be stored orthogonally, especially in the face of interference. Mnemonic discrimination tasks have been designed to tax hippocampal pattern separation by including perceptually similar “lure” stimuli during memory retrieval. However, we experience vast interference beyond a single instance of overlap. Thus, a key feature of our memory system is to overcome this high interference. Furthermore, some experiences tend to be better remembered by most people than others, a feature known as memorability. However, it is unclear how memorability may impact the effect of interference on memory. To this end, we designed a mnemonic discrimination task with multiple forms of interference, such that target (repeated) and lure (similar) images of a baseline image were shown to participants during a memory test designed to increase interference during memory retrieval. We additionally varied image memorability by including memorable and forgettable images to examine interactions with interference conditions. We found that greater interference during retrieval enhanced lure discrimination for memorable images, but impaired lure discrimination for forgettable images. This suggests that interference does not uniformly impact memory, with greater interference in memory leading to exaggerated memorable and forgettable experiences.

## 1 Introduction

We often do the same things day after day—we eat breakfast, lock our front door, commute to work, talk to many of the same people, and usually take the same route home. This overlap across our daily experiences can make differentiating one experience from another more difficult (i.e., where you parked your car today versus yesterday). Experiences that share features can impact one's ability to accurately recognize them as independent (Reagh and Yassa 2014b). Overcoming interference across similar experiences is an essential feature of a successful memory system. Computational models propose that we can remember similar events as unique experiences by balancing two key computations performed by the hippocampus: pattern separation and pattern completion (Leal and Yassa 2015). Pattern completion refers to the ability to accurately generalize information when given partial cues (Larocque et al. 2013), while pattern separation refers to the ability to accurately discriminate among similar experiences, allowing them to be stored as distinct representations (Yassa and Stark 2011). For instance, recognizing your friend, even though they got a new haircut, would be an example of pattern completion. However, noticing that your friend cut their hair, even if it was just an inch or so, would be an example of successful pattern separation. Pattern separation relies on the hippocampal subfield dentate gyrus (DG) and its mossy fiber connections to hippocampal subfield CA3 (Larocque et al. 2013).

To tax hippocampal pattern separation in humans, mnemonic discrimination tasks (MDTs) have been developed, which provide a behavioral correlate of hippocampal pattern separation by including similar “lure” stimuli during retrieval (Leal and Yassa 2018; S. M. Stark et al. 2019). MDTs allow both general recognition and lure discrimination (the behavioral correlate of pattern separation) to be measured by including repeated stimuli during retrieval (targets) as well as similar stimuli (lures), respectively. Such tasks have become popular tests of memory and hippocampal function given their sensitivity to clinical conditions with hippocampal dysfunction (Leal and Yassa 2018) and have been developed across many domains of memory including spatial, temporal, face‐name, and emotional variants (Leal, Tighe, and Yassa 2014; Mannion et al. 2023; Reagh et al. 2014). This paradigm is based on the idea that when undifferentiated traces occur during encoding, the inability to separate overlapping representations culminates in interference and forgetting (McClelland et al. 1995).

Multiple Trace Theory (MTT) hypothesizes that a new trace within the hippocampus is formed every time a memory is reactivated. In this model, when older memories are reactivated, they form more traces, making them more resistant to disruption (Moscovitch and Gilboa 2021; Nadel et al. 2000). However, the temporal gradient for this model is flat, where if the hippocampus is completely damaged, both recent and remote memories are lost. On the other hand, competitive trace theory (CTT) (Yassa and Reagh 2013) presents a continuum model, in which the hippocampus' central role during retrieval is reconstructing memories using overlapping traces. CTT posits that as memories get older, they become more decontextualized due to competition among overlapping representations, becoming more reliant on storage within the neocortex. In relation to hippocampal pattern separation and completion, CTT states that hippocampal traces are nonoverlapping due to pattern separation, while in the neocortex, overlapping representations are strengthened.

Interference effects in episodic memory have traditionally been studied within an MDT framework, where performance is known to decline with increasing lure similarity. Interference theories such as CTT propose that multiple exposures to overlapping or conceptually related stimuli, either during encoding or retrieval, can also lead to competition among memory traces, where each reactivation provides an opportunity for both strengthening and distortion, depending on the extent of overlap. It is important to note, however, that real‐world interference is far more complex and variable. Creating laboratory tasks that mimic a more naturalistic retrieval effort and capture the complexities of remembering everyday experiences can generate more ecologically valid memory measures. Disambiguating overlapping experiences and overcoming interference is a critical feature of daily life; thus, approximating these complexities in experimental settings can provide us with paradigms to better understand and measure memory function.

One feature that may contribute to the influence of interference on memory is the memorability of the events to be remembered, or the tendency for certain experiences to be better remembered by most people (Rust and Mehrpour 2020). Memorability represents a measurable and intrinsic quality shared among various stimuli such as faces, words, images, artwork, and movements (Bainbridge et al. 2013; Davis and Bainbridge 2023; Goetschalckx and Wagemans 2019; Isola et al. 2014). Notably, memorable stimuli exhibit a high degree of consistency in being remembered across individuals and remain largely unaffected by time and contextual factors (Goetschalckx et al. 2019, 2017). Research has shown that memorability operates independently of traditional memory facilitators like attention (e.g., depth of processing, automatic attentional capture) and priming effects (e.g., directed forgetting, repetition) (Bainbridge 2020; Mancas and Le Meur 2013). Studies examining the neural correlates of image memorability have shown that perirhinal and parahippocampal cortices within the medial temporal lobe (MTL) are particularly sensitive to the memorability of a stimulus, showing representational organization (Bainbridge and Rissman 2018). Despite the intrinsic nature of memorability, its precise influence on information encoding and storage remains unclear. While some theories suggest that hippocampal pattern completion and separation computations may elucidate variations in memorability (Akagunduz et al. 2020; Kramer et al. 2023), these two areas of research have largely operated independently. Notably, assessment and quantification of memorability has predominantly relied on testing recognition accuracy by using conventional recognition memory tasks (Isola et al. 2014, 2011), where repeated and novel stimuli are presented to participants who need to correctly recognize these repeated targets as “old.” The memorability of an item is then defined by its mean hit rate (correctly identifying repeated images as “old”) minus the correct rejection (CR) rate (correctly identifying novel images as “new”). While useful, recognition tasks neglect the intricate processes involved in encoding and retrieving information in memory, namely interference.

Previous studies have employed MDTs while taking memorability into account and have found that while effects of memorability (e.g., memorable > forgettable) were present for target recognition, the impact of memorability on lure discrimination only benefited lower similarity items, which tend to be easier to discriminate (Morales‐Calva et al. 2025). Furthermore, these effects have been shown to be impacted by the emotional content of the images, where lure discrimination for emotional images was hindered in comparison to neutral images, and forgettable neutral images were better discriminated compared to memorable neutral images (Morales‐Calva and Leal 2024). However, while emotional valence and arousal contribute to memorability, they cannot fully explain its effects (Wakeland‐Hart and Aly 2025). Recent work has examined how memorability benefits emerge within visual working memory and extend into long‐term memory (Gillies et al. 2023). They tested three hypotheses: (1) the efficiency hypothesis (e.g., memorable stimuli may be preferentially encoded into memory because fewer cognitive resources are required to store them in visual working memory, which is capacity‐limited); (2) the competitiveness hypothesis (e.g., memorable stimuli may be more competitive in obtaining cognitive resources than forgettable stimuli); and (3) the stickiness hypothesis (e.g., memorable stimuli may be less prone to be forgotten than forgettable stimuli after they pass through the encoding bottleneck). They found that people remembered memorable images better than forgettable ones, both when viewing them alone and when competing for attention in a visual working memory task. However, only the efficiency advantage led to better long‐term memory performance. Once memorable images made it into long‐term memory, they were also less likely to be forgotten. This study suggests that memorability boosts memory at several stages of processing. Nonetheless, it is unclear how interference during retrieval may impact memory of memorable and forgetting images. While mnemonic discrimination paradigms induce interference during retrieval through the inclusion of perceptually similar lure stimuli, repetition during retrieval is another way to induce interference.

Repetition is often thought to enhance memory (Xue et al. 2010), as we often do when studying for an exam or trying to remember a phone number. In some cases, repetition can make representations more resistant to forgetting, as it can reduce the attentional resources needed for cognitive processing (Moscovitch and Gilboa 2021; Nadel et al. 2000). Repetition may support the reinforcement of neural pathways associated with particular events as well as in the strengthening of connections between existing information, fostering familiarity and generalization, and facilitating the retention of information for the long term (Tulving and Schacter 1990). However, it has been hypothesized that repetition elicits a similar but nonidentical memory trace, and that contextual details of traces may compete for representation over time (Yassa and Reagh 2013). Thus, repetition may not enhance memory in all cases. Multiple traces of the same event can create interference and reduce attention to subsequent presentations, hence decreasing the likelihood that the associations between novel and existing representations will be encoded (Kim et al. 2012). Studies using MDTs have shown that stimulus repetition during encoding benefits recognition memory but impairs lure discrimination (Reagh and Yassa 2014b). However, this may depend on how memory is measured, as a similar study applying a different framework for analyzing the same memory measures yielded opposite findings, with lure discrimination improving with repeated encoding (Loiotile and Courtney 2015).

Memorability has been shown to be independent of repetition priming, in which viewing an image repeatedly does not increase an image's memorability (Bainbridge 2020). A recent study investigated the varying effects of memorability across experimental paradigms by presenting up to 10 semantically similar exemplars during encoding, which were later repeated during retrieval alongside novel, unrelated exemplars (Zhao et al. 2024). Although the similarity of repeated items between encoding and retrieval could have been leveraged to behaviorally assess pattern separation, the stimuli were instead used solely to measure target repetition and novel item rejection. Thus, it is still unclear whether repetition is facilitatory or if it creates interference in memory, as opposing views have been found in multiple paradigms. Repetition may bias how that information gets processed and stored.

Furthermore, while memory tasks commonly used in neuropsychological batteries and laboratory experiments test participants within the same day, most of our everyday life memory problems result from longer delays between encoding and retrieval (Leal et al. 2019). The degree of forgetting depends on the length of the delay, the type of information being remembered, and other factors that can influence how memories are stored (Kensinger et al. 2007; Leal and Yassa 2014). Memory performance typically declines with increased retention intervals and would also be expected of behavioral memorability scores, which have been stated to decline log‐linearly (Khosla et al. 2015). Memorability impacts are stable shortly after encoding and could be resistant to forgetting over time; however, most studies have examined differences between ~5 and 40 min and not at extended time periods for retention intervals (Goetschalckx et al. 2017). Previous research has shown that both target recognition and lure discrimination performance is worse after a 24‐h delay across young and older adults (Leal et al. 2019). Moreover, when looking at the interaction of memorability and time delays in a mnemonic discrimination paradigm, we have shown that memorable images are better remembered across both target recognition and lure discrimination measures; however, for target recognition, this effect was not observed after a 24‐h delay (Morales‐Calva and Leal 2024).

In the current study, we aimed to test whether the effects of memorability were resilient to greater levels of interference induced by including both repeated and lure images during retrieval in a MDT. The present study builds on three theoretical frameworks: (1) Mnemonic Discrimination/Pattern Separation, (2) Competitive Trace Theory, and (3) Memorability, where interference was introduced in two ways: (1) by including similar lure stimuli, as is typical of MDTs, where test items share overlapping features with baseline stimuli, and (2) by including both lures *and* targets during retrieval, introducing multiple traces that our memory system must resolve. It is also important to consider how initial retrieval (of either a target or a lure) and its associated response influence subsequent memory for the second exposure. Thus, greater interference arises not only from repeated or similar exposures to baseline images but also from how responses to the first exposure affect responses to the second. Importantly, including both targets and lures during retrieval may increase competition among overlapping traces while also providing opportunities for re‐encoding both stimulus features and behavioral responses. Our design, therefore, captures both retrieval‐based interference, where similar traces compete during memory decisions, and response‐dependent re‐encoding, where responses to earlier trials influence subsequent retrieval. This interplay between retrieval and re‐encoding aligns with frameworks emphasizing competition and updating during repeated retrieval (Kim et al. 2012; Yassa and Reagh 2013).

Participants underwent either a standard MDT that included either a lure *or* a repeated image during retrieval of an image shown during encoding, or the modified version of the standard MDT, which included both the target *and* the lure of an image shown during encoding (baseline image). The presentation order of the target and lure during retrieval was randomized, such that sometimes the target (repeated) image appeared first (target first, lure second), and sometimes the lure (similar) image appeared first (lure first, target second). The number of images between the presentation of first and second paired images was randomized. Thus, as a secondary aim, we sought to determine whether presentation order as well as memory on the first stimulus of the pair during retrieval influenced subsequent memory. We aimed to increase interference by including similar lure images during retrieval in addition to exposing participants to multiple related stimuli during retrieval (target and lures), creating more overlap across events within the task, with the objective of more closely mimicking the interference that occurs in everyday life. Finally, to emulate naturalistic retrieval efforts, we also included a 24‐h delay, as most forgetting tends to occur after 24 h (Leal et al. 2019; Morales‐Calva and Leal 2025).

First, we hypothesized that this manipulation of including both target and lure images during retrieval of an image shown during encoding, would lead to worse memory performance due to increased interference (both target and lure of a baseline image presented during retrieval) compared to standard MDT performance (either target or lure of a baseline image presented during retrieval), independent of presentation order and accuracy on the first stimulus of the pair. Second, we hypothesized that memorability would impact these effects on memory, such that memorable images would not be as susceptible to the effects of interference compared to forgettable images. Third, we hypothesized that memorable images would be more resilient to the effects of extended time delay compared to forgettable images, thus showing differential effects of interference across these variables. Fourth, based on the idea of repeated exposures causing greater interference, we predicted that first presentations (target first—T1, lure first—L1) would be better remembered than second presentations (target second—T2, lure second—L2) for both lure and target images; given that first presentations are replicating traditional MDTs and have experienced less interference than the second paired image. Finally, we predicted that correctly identifying the first stimulus (T1 or L1) would enhance subsequent memory of the second paired stimulus (L2 or T2). On the other hand, we predicted that incorrect recognition of a first presentation (T1 or L1) could lead to increases in false alarms for their paired presentations.

## 2 Materials and Methods

### 2.1 Participants

In Experiment 1, participants completed a standard MDT that has been balanced across memorable and forgettable images, results of which were recently published (Morales‐Calva et al. 2025). Young adults (*N* = 52, ages 18–35) were recruited from the Houston community through listservs, website postings, and flyers. Participants were compensated with gift cards or cash for their participation in the study. All participants were fluent in English and had normal or corrected to normal vision. Participant demographic details can be found in Table 1. Twenty‐six participants were tested immediately following encoding and 26 were tested following a 24‐h delay.

**TABLE 1:** Participant demographics for Experiment 1: standard MDT.

Variable	Immediate	Delay	Total
*N*	26	26	52
Age	19.2 ± 1.1	21 ± 3.2	20.1 ± 2.5
Years of education	12.6 ± 1.6	14.5 ± 2.8	13.5 ± 2.5
Gender M:F:NBi	11:14:1	12:14:0	23:28:1

Race & ethnicity		
	Non‐Latinx/o	Latinx/o
MENA	1	0
Asian	21	0
Asian & Pacific Islander	1	0
Asian & White	2	1
Black	3	1
Black & White	1	1
White	14	5
White & Indigenous	0	1

For Experiment 2, a new sample of participants performed a modified version of the MDT, with both target *and* lure images shown during retrieval. Young adults (*N* = 50, ages 18–35) were recruited from the Houston community through listservs, website postings, and flyers. Participants were compensated with gift cards or cash for their participation in the study. All participants were fluent in English and had normal or corrected to normal vision. Participant demographic details for Experiment 2 can be found in Table 2. Twenty‐five participants were tested immediately following encoding, and 25 participants were tested following a 24‐h delay. All participants in Experiment 2 were independent from participants in Experiment 1 to circumvent previous experience with the stimuli. All participants provided informed consent to participate in the study, with all experimental protocols approved by the Rice University Institutional Review Board (Protocol Number IRB‐FY2020‐6; Title: Cognitive, MRI, and PET studies of memory systems across the lifespan).

**TABLE 2:** Participant demographics for Experiment 2: modified MDT.

Variable	Immediate	Delay	Total
*N*	25	25	50
Age	22.2 ± 3.6	23.4 ± 4.7	22.8 ± 4.2
Years of education	15.6 ± 3.3	15.3 ± 2.4	15.5 ± 2.8
Gender M:F:NBi	7:16:2	8:17:0	15:33:2

Race & ethnicity		
	Non‐Latino	Latino
Indigenous	0	1
Indigenous & White	0	3
MENA & White	1	0
Asian	15	0
Black	5	1
White	18	6

### 2.2 Memory Tasks

#### 2.2.1 Experiment 1: Memorability MDT (Standard Version)

**FIGURE 1:**
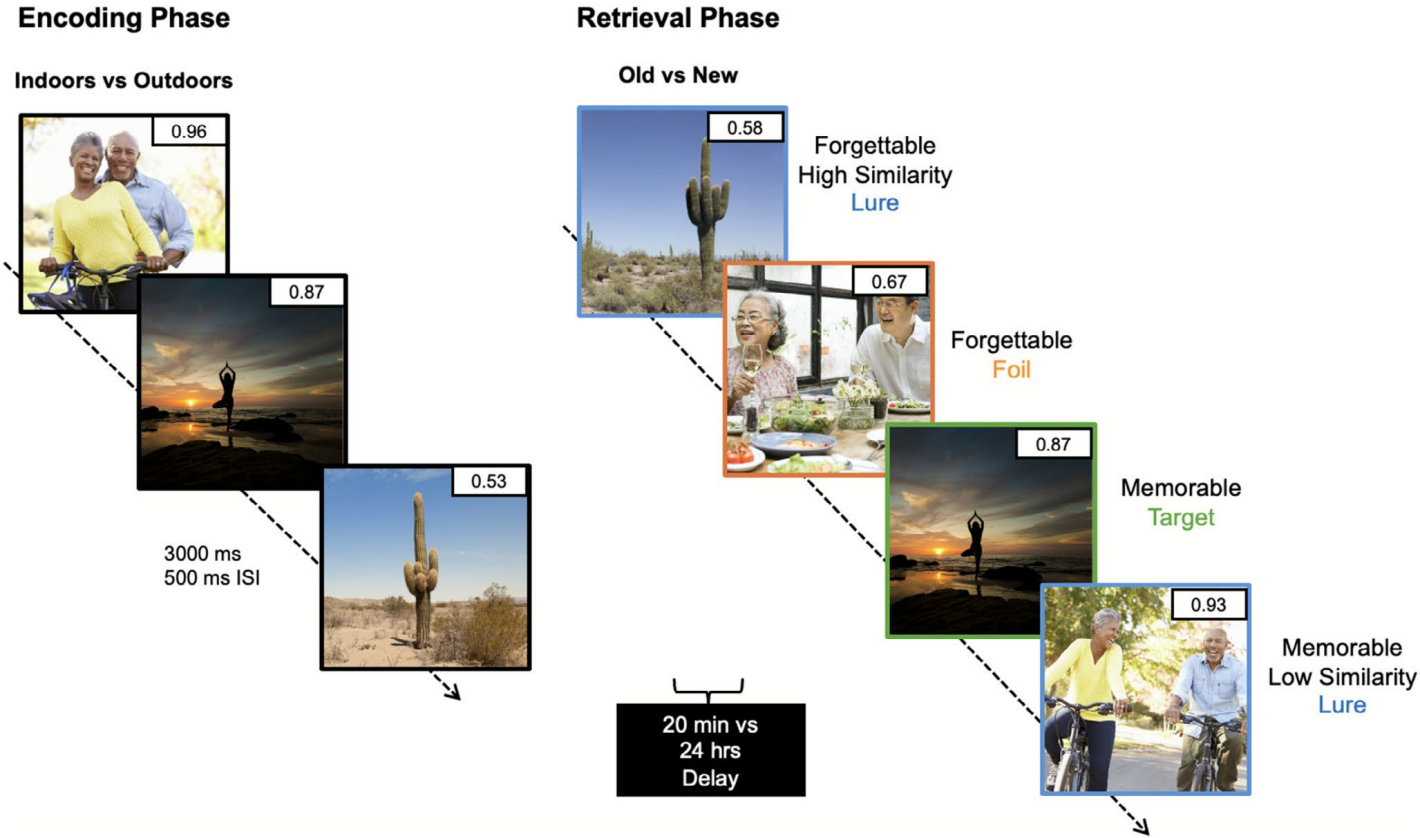
Standard mnemonic discrimination task balanced across memorability and similarity. During the Encoding Phase, participants were shown a series of images (3000 ms stimulus presentation, 500 ms inter‐stimulus interval; ISI) and were asked to identify them as “indoors” or “outdoors.” After 20‐min (immediate) or 24‐h (delay) during the Retrieval Phase, participants were given a surprise memory test, in which images were either repeated (target = green), similar, but not identical (lure = blue), or a brand new image (foil = orange), and had to respond whether the image was “old” or “new.”

Participants completed a standard MDT including images characterized for memorability (e.g., memorable and forgettable; Figure 1). The task included images quantified on memorability from the *MemCat* dataset (Goetschalckx and Wagemans 2019) and varied in image memorability (0.2–0.9) and image similarity (low, medium, high). Image similarity scores were determined through ratings on a scale from 1 to 7 (1 = unrelated, 3 = low similarity, 5 = high similarity, 7 = exact match) (see stimulus development in Morales‐Calva et al. (2025) for full stimuli selection and task development details). During the encoding phase, participants were shown 160 images and were asked to categorize them as either belonging more “indoors” or “outdoors,” as an attentional check. The images were shown one at a time in randomized order for 3000 ms with a 500 ms ISI (~10 min). After the encoding phase, participants completed a series of questionnaires lasting 1 h. Then, half the participants were tested on their memory for the images in the retrieval phase (immediate group) and the other half were tested 24 h later (delay group). During retrieval, participants were shown 240 images in randomized order, with some images being completely novel (foils), some being repeated images those shown during encoding (targets), and some images being similar, but not identical, to images shown during encoding (lures). Participants were asked to identify the images as either “old” if the image was exactly the same as one shown during encoding, or “new” if it was novel or similar, but not exactly the same, as those seen during encoding. The retrieval phase included a break for participants halfway through the task to minimize fatigue. Each section of the retrieval phase took ~7 min, for a total of ~14 min of total retrieval phase duration. After retrieval, both participant groups were asked to complete a series of questionnaires (see Supporting Information 1 in Data S1).

#### 2.2.2 Experiment 2: Memorability MDT (Modified Version)

**FIGURE 2:**
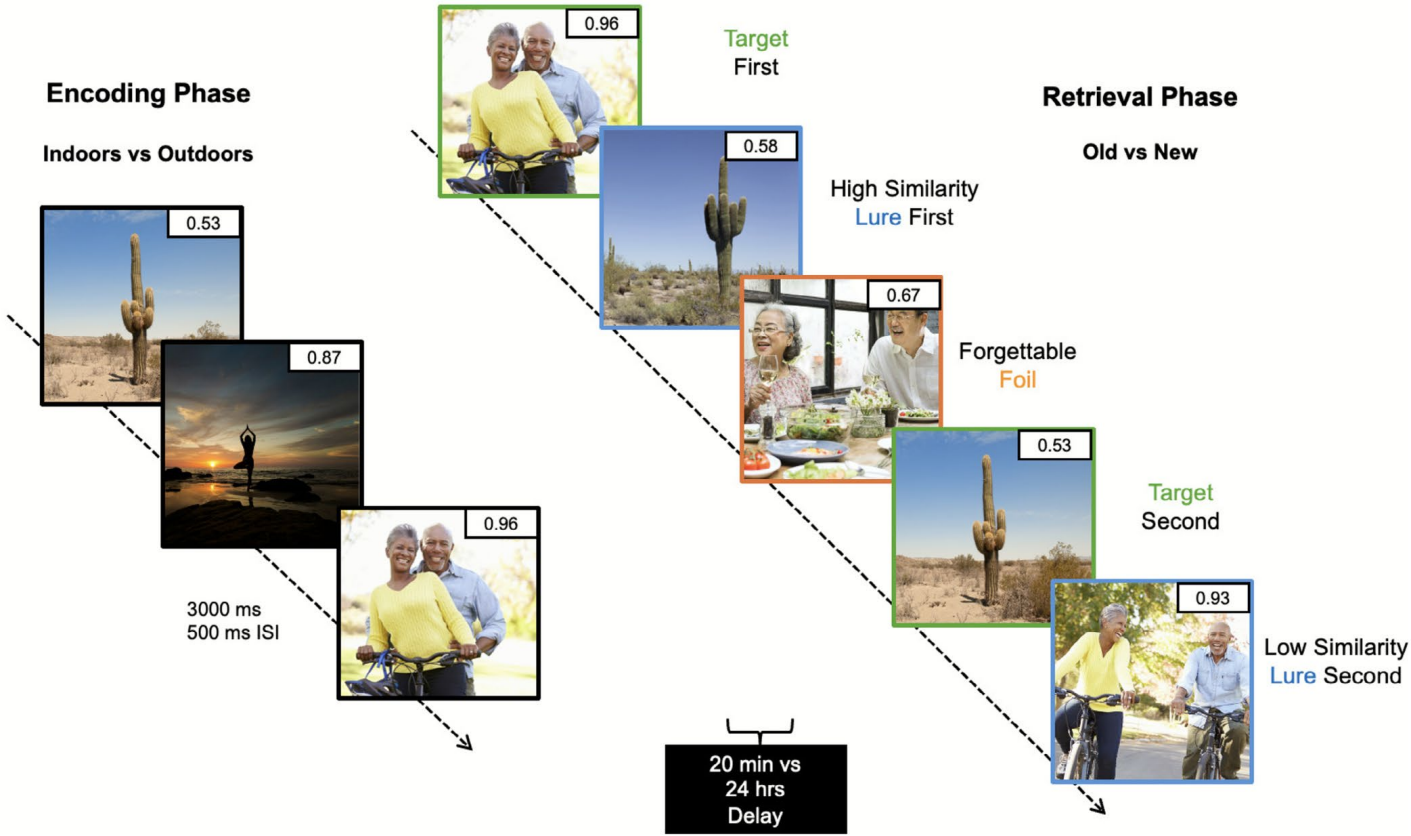
Modified mnemonic discrimination task with increased inference balanced across memorability and similarity. During the Encoding Phase, participants were shown a series of images (3000 ms stimulus presentation, 500 ms inter‐stimulus interval; ISI) and were asked to identify them as “indoors” or “outdoors.” After 20‐min (immediate) or 24‐h (delay) during the Retrieval Phase, participants were given a surprise memory test. Retrieval images included both a repeated image (target = green) and a similar, but not identical image (lure = blue) for each corresponding baseline image shown during encoding or were shown a new image (foil = orange) and had to respond whether the image was “old” or “new.” Presentation order of targets and lures were randomly shown either first or second in the series of images.

A different group of participants was tested on a modified version of the MDT described in Experiment 1. The task included the same images and instructions as in Experiment 1, however, to try to further increase interference in memory, both the target (repeated image) of an image shown during encoding, *and* the lure (similar image) of the same baseline image, were shown during retrieval (Figure 2). Paired stimuli conditions were presented randomly throughout the retrieval phase so that the lag between first and second presentations differed across paired items and to avoid order bias; hence not all participants were presented with the same presentation order of conditions across the same stimulus set. Participants were either shown a repeated image first (target first = T1) and were later shown the corresponding lure image (lure second = L2); or were shown the lure image first (lure first = L1) and were later shown the corresponding target image (target second = T2). When the target image was shown first during retrieval (T1 e.g., couple posing in Figure 2), we hypothesized that this initial exposure to the target image would avoid interference with the lure and would strengthen the memory trace for the target image. However, this could, in turn, negatively impact lure discrimination of its corresponding lure image shown second, due to greater interference (L2) (Reagh and Yassa 2014b). When the lure image was shown first (L1, e.g., cactus in Figure 2), the participant would need to rely on pattern separation to discriminate the lure. However, this could negatively impact target recognition of its corresponding target image shown second (T2, increased interference). These predictions may also depend on whether participants are accurately recognizing or discriminating the “first” images of the pair (T1 or L1), which may then impact their ability to accurately recognize or discriminate the “second” images of the pair (T2 or L2). Correct recognition or discrimination of the first image might enhance performance on the second image (T2 or L2), whereas errors on the first presentation could lead to greater interference and further impair accuracy on the second image. Participants did not receive any feedback on their responses during retrieval, ensuring that response‐dependent effects were internally generated rather than guided by external correction. The stimuli were balanced across memorability and similarity; stimuli variety across retrieval and encoding phases can be found in Supporting Information 2 in Data S1.

#### 2.2.3 Memory Measures of Interest

Across both experiments, we examined two key memory measures of interest: *lure discrimination* and *target recognition*. Based on signal detection theory (Yonelinas et al. 2010), target recognition was measured by a discriminability index (*d*′) defined as the difference between the normalized proportion of target (old) stimuli judged as “old” (hit) and the normalized proportion of foils (novel) stimuli judged as “old” (false alarm); which was calculated as [*z*(*p*(‘Old’|Target)) − *z*(*p*(‘Old’|Foil))]. Lure discrimination index (LDI), which provides a behavioral correlate of hippocampal pattern separation (S. M. Stark et al. 2019), was calculated by the proportion of “new” responses to a lure (correct rejection), and correcting for response bias by subtracting out the proportion of “new” responses, to a target stimulus (miss), calculated as [*p*(‘New’|Lure) − *p*(‘New’|Target)]. These measures were calculated across levels of memorability (memorable, forgettable), levels of lure similarity (low, medium, high), and presentation order (first, second) (see Supporting Information 3 in Data S1 for a visual description of levels of analysis). Raw proportions of correct responses can be found in Supporting Information 4 in Data S1.

### 2.3 Statistical Analyses

All statistical analyses were conducted using JASP 0.16.2 (JASP Team 2022) statistical software. All data were examined for outliers where any observation more than three standard deviations from the sample mean was considered an outlier. Data from one participant in the immediate group of Experiment 2 were excluded after being identified as an outlier and excluded from the analyses looking at the interaction between similarity and order presentation in LDI and, consequently, when looking at the interaction between similarity, presentation order, and memorability in LDI. Data from a different participant in the immediate group of Experiment 2 were excluded when looking at the interaction between memorability and group in *d*′ performance as it was identified as an outlier. Student's *t*‐test was used to determine the difference between categories and groups, when Levene's test of equality of variances was significant, Welch's *t*‐test was performed. Multiple comparisons were analyzed through simple and repeated‐measures ANOVAs. When repeated‐measures tests violated the sphericity assumption as measured by Mauchly's test, Greenhouse–Geisser correction was used. To determine the strength of the reported relationships Cohen's *d* and partial eta squared ($\eta_{p}^{2}$) are reported for effect sizes. *Post hoc* contrasts were conducted using Bonferroni correction or Scheffé's method in SPSS (IBM Corp 2022), where appropriate. Statistical values were considered significant at a final corrected alpha level of 0.05, which controlled for Type I error. Sample sizes are in line with previous mnemonic discrimination paradigms (Leal et al. 2019; Leal, Tighe, and Yassa 2014; Phillips et al. 2023; Reagh and Yassa 2014a), but we also conducted *post hoc* effect size sensitivity analyses using G*Power 3.1 (Faul et al. 2007) to explore the effect sizes we could detect with 80% power given our sample size and alpha of 0.05. For our analyses of variance and difference between means, we were powered to detect small to medium effects across groups (*d* = 0.27, *f* = 0.14) (Cohen 1992, 2009).

## 3 Results

### 3.1 Replication of Established Mnemonic Discrimination and Memorability Trends in Modified MDT

**FIGURE 3:**
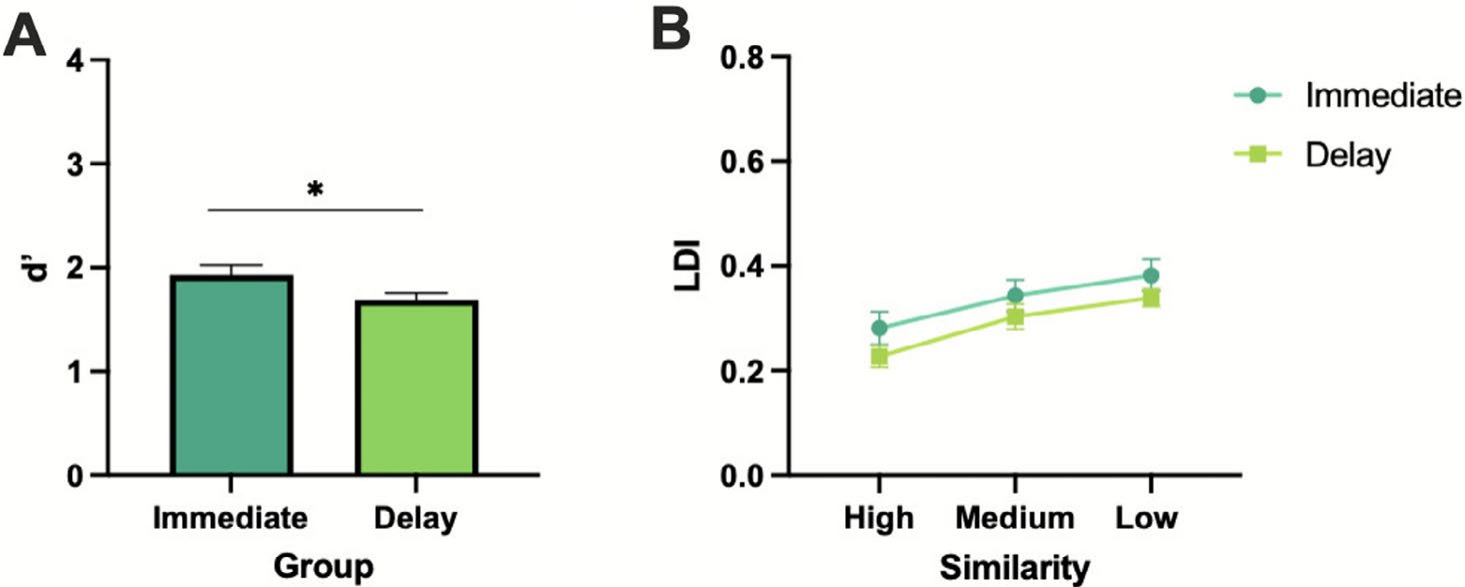
Replication of effects from mnemonic discrimination tasks. Differences in average target recognition (*d*′) (A) and lure discrimination index (LDI) (B) across immediate and delay testing times. Immediate *N* = 25, delay *N* = 25. Error bars represent SEM. *p ≤ 0.05.

First, we examined the overall results from *d*′ and LDI on the modified MDT to determine whether our task manipulation (i.e., modified version) would replicate findings of well‐established factors (e.g., time of testing, lure similarity) on mnemonic discrimination (Lacy et al. 2011; Leal and Yassa 2014). We examined differences in *d*′ across immediate and delay groups using an independent‐samples *t*‐test. As expected, there was a statistically significant difference in target recognition across groups [*t*(48) = 2.05, *p* = 0.04, *d* = 0.58; Figure 3A] with the immediate group showing higher *d*′ than the delay group. For lure discrimination, we conducted a repeated‐measures ANOVA with similarity (high, medium, low) as the within‐subjects factor and group (immediate, delay) as the between‐subjects factor. There was a main effect of similarity [*F*(2,96) = 45.65, *p* < 0.001, $\eta_{p}^{2}=0.48$], where low‐similarity items were easiest to discriminate, followed by medium, and finally by high similarity [*F*(1,48) = 76.52, *p* < 0.001, $\eta_{p}^{2}=0.62$; Figure 3B]. There was no main effect of group (*p* = 0.19) nor an interaction between similarity and group (*p* = 0.801).

**FIGURE 4:**
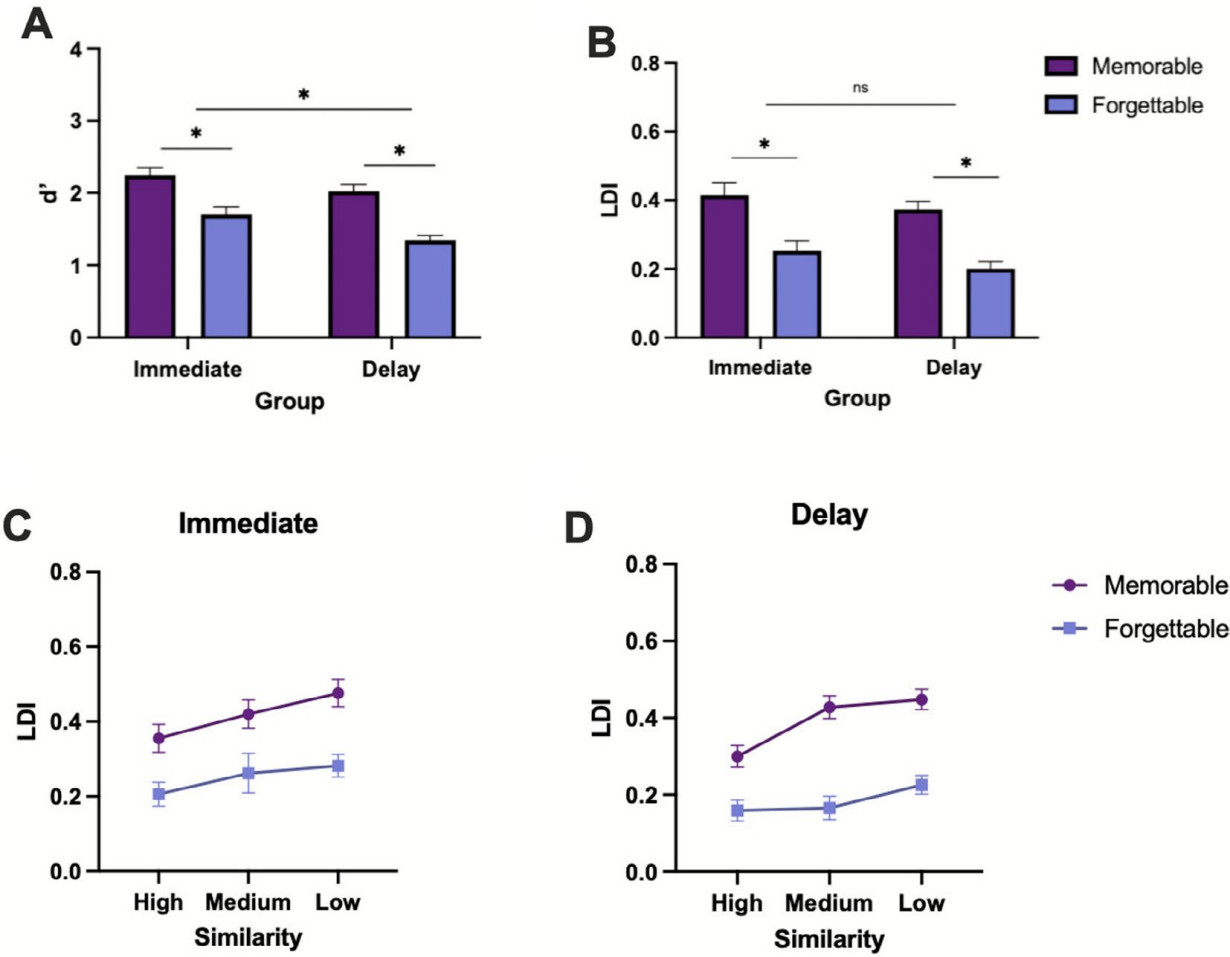
Performance on target recognition and lure discrimination across memorability at different time delays. Differences in average (A) target recognition (*d*′) and (B) lure discrimination index (LDI) at immediate and delay testing times for memorable and forgettable images. LDI across three levels of similarity (high, medium, low) during (C) immediate memory performance and (D) delayed memory performance. Error bars represent SEM. *p ≤ 0.05. Immediate *N* = 25, delay *N* = 25.

Next, to analyze the effects of memorability on target recognition, we performed a repeated‐measures ANOVA with memorability (memorable, forgettable) as the within‐subjects factor and group (immediate, delay) as the between‐subjects factor. We found that memorable items were better remembered than forgettable ones [*F*(1,47) = 69.58, *p* < 0.001, $\eta_{p}^{2}=0.597$], as expected. We also found a main effect of group, in which those who were tested immediately outperformed those tested after 24‐h [*F*(1,47) = 7.64, *p* = 0.008, $\eta_{p}^{2}=0.140$], also as expected (Figure 4A). There was no significant interaction between memorability and group (*p* = 0.36). For lure discrimination, we conducted a repeated‐measures ANOVA with memorability (memorable, forgettable) as the within‐subjects factor and group (immediate, delay) as the between‐subjects factor. We found a significant effect of memorability [*F*(1,48) = 87.34, *p* < 0.001, $\eta_{p}^{2}=0.645$], in which memorable images were better discriminated than forgettable images. There was no main effect of group (*p* = 0.18), nor a significant interaction between memorability and group (*p* = 0.09) (Figure 4B).

To analyze the interaction between memorability and lure similarity, we conducted a repeated‐measures ANOVA with memorability (memorable, forgettable) and similarity (high, medium, low) as within‐subjects factors within each group. In the immediate group, we found a significant main effect of similarity [*F*(2,48) = 23.812, *p* < 0.001, $\eta_{p}^{2}=0.49$; Figure 4C], with low similarity, followed by medium, then high similarity being most difficult to discriminate [*F*(1,24) = 45.78, *p* < 0.001, $\eta_{p}^{2}=0.65$]. We also found a main effect of memorability [*F*(1,24) = 61.15, *p* < 0.001, $\eta_{p}^{2}=0.72$], where memorable images were better discriminated than their forgettable counterparts. There was no significant interaction between memorability and similarity [*F*(2,48) = 0.61, *p* = 0.51, $\eta_{p}^{2}=0.025$]. In the delay group, we also found a significant effect of similarity [*F*(2,48) = 19.65, *p* < 0.001, $\eta_{p}^{2}=0.45$; Figure 4D] with low similarity, followed by medium, then high similarity being most difficult to discriminate [*F*(1,24) = 30.69, *p* < 0.001, $\eta_{p}^{2}=0.56$]. There was a significant effect of memorability [*F*(1,24) = 66.62, *p* < 0.001, $\eta_{p}^{2}=0.735$], with forgettable items yielding worse LDI than memorable images. Only in the delayed group did we find a significant interaction between memorability and similarity [*F*(2,48) = 3.57, *p* = 0.036, $\eta_{p}^{2}=0.13$], where medium similarity images showed the largest difference between memorable versus forgettable lure discrimination compared to low and high similarity images [*F*(1,24) = 4.89, *p* = 0.037, $\eta_{p}^{2}=0.17$]. When conducting a 3‐way ANOVA also including group as a factor (immediate, delay), all results remained the same except the interaction between memorability and similarity became marginal [*F*(2,96) = 2.77, *p* = 0.07, $\eta_{p}^{2}=0.03$]. There was no main effect of group [*F*(1,48) = 1.75, *p* = 0.19, $\eta_{p}^{2}=0.04$] nor any significant interactions between group and memorability (*p* = 0.21), group and similarity (*p* = 0.91), and memorability, similarity, and group (*p* = 0.20).

### 3.2 Greater Interference Exaggerates the Effects of Memorability on Lure Discrimination

**FIGURE 5:**
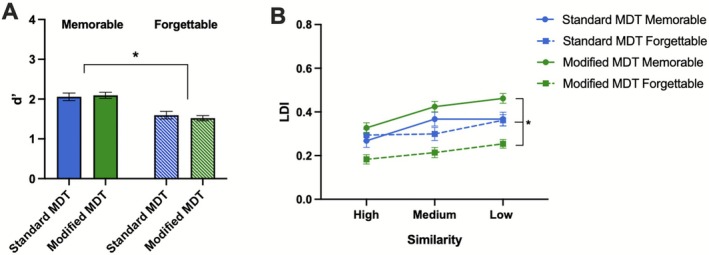
The impact of increased interference from multiple exposures on memorable and forgettable images on target recognition and lure discrimination. (A) Target recognition (*d*′) performance for memorable and forgettable images on standard and modified mnemonic discrimination tasks (MDT). (B) Lure discrimination index (LDI) performance for memorable and forgettable images on standard and modified MDTs. Error bars represent SEM. *p ≤ 0.05. Immediate *N* = 25, delay *N* = 25.

To determine how the inclusion of both target and lure stimuli during retrieval impacted memory in our modified MDT relative to the standard MDT, we compared memory performance across Experiment 1 (standard version—either lure *or* target during retrieval) and Experiment 2 (modified version—both lure *and* target during retrieval). For ease of interpretation given that time effects were expected, we collapsed across time delays but also included results from the 4‐way ANOVA (see Supporting Information 5 and 6 in Data S1). For target recognition, we conducted a repeated‐measures ANOVA with memorability (memorable, forgettable) as the within‐subjects factor and task (standard, modified) as a between‐subjects factor. There was a main effect of memorability [*F*(1,100) = 88.41, *p* < 0.001, $\eta_{p}^{2}=0.46$; Figure 5A], with memorable images being better remembered than forgettable ones. There were no main effects of task (*p* = 0.85) nor a significant interaction between memorability and task (*p* = 0.30). When conducting the 4‐way ANOVA with group (immediate, delay) included as an additional between‐subjects factor, the only additional effect was the expected significant main effect of group, where those tested immediately showed better target recognition than those tested after 24 h [*F*(1,98) = 11.9, *p* < 0.001, $\eta_{p}^{2}=0.109$; Figure S5 in Data S1].

For lure discrimination, we conducted a repeated‐measures ANOVA with memorability (memorable, forgettable) and similarity (low, medium, high) as the within‐subjects factor and task (standard, modified) as the between‐subjects factor. We found a main effect of memorability [*F*(1,100) = 92.0, *p* < 0.001, $\eta_{p}^{2}=0.48$], with memorable images being better discriminated than forgettable images. There was a main effect of similarity [*F*(2,200) = 84.08, *p* < 0.001, $\eta_{p}^{2}=0.46$], with better lure discrimination as lure similarity decreased [*F*(1,100) = 174.48, *p* < 0.001, $\eta_{p}^{2}=0.64$]. Finally, there was a significant interaction between similarity and memorability [*F*(2,200) = 9.87, *p* < 0.001, $\eta_{p}^{2}=0.09$], where memorable images showed better lure discrimination as lure similarity decreased, while forgettable images showed no difference in lure discrimination across similarity levels [*F*(1,100) = 21.82, *p* < 0.001, $\eta_{p}^{2}=0.17$]. There was no main effect of task (*p* = 0.322), but there was a significant interaction between memorability and task, in which there was a larger difference in lure discrimination for memorable versus forgettable images on the modified increased inference task compared to the standard task [*F*(1,100) = 33.84, *p* < 0.001, $\eta_{p}^{2}=0.25$; Figure 5B]. Interestingly, memorable images from the modified increased interference task were better remembered compared to standard task performance, and the opposite was true for forgettable images, where the increased interference task showed worse lure discrimination for forgettable images compared to standard task performance. The interactions between similarity and task (*p* = 0.10) and memorability, similarity, and task (*p* = 0.43) were not statistically significant. When we conducted the 4‐way ANOVA with group (immediate, delay) included, we found the same results described above in addition to the expected effect of group [*F*(1,98) = 13.8, *p* < 0.001, $\eta_{p}^{2}=0.123$], in which participants tested immediately outperformed those tested after 24 h. We also found a significant interaction between task and time [*F*(1,98) = 4.48, *p* = 0.037, $\eta_{p}^{2}=0.044$; Figure S6 in Data S1], with larger differences shown between immediate and delayed testing in the standard task than in the modified task.

### 3.3 No Effect of Presentation Order on Performance

**FIGURE 6:**
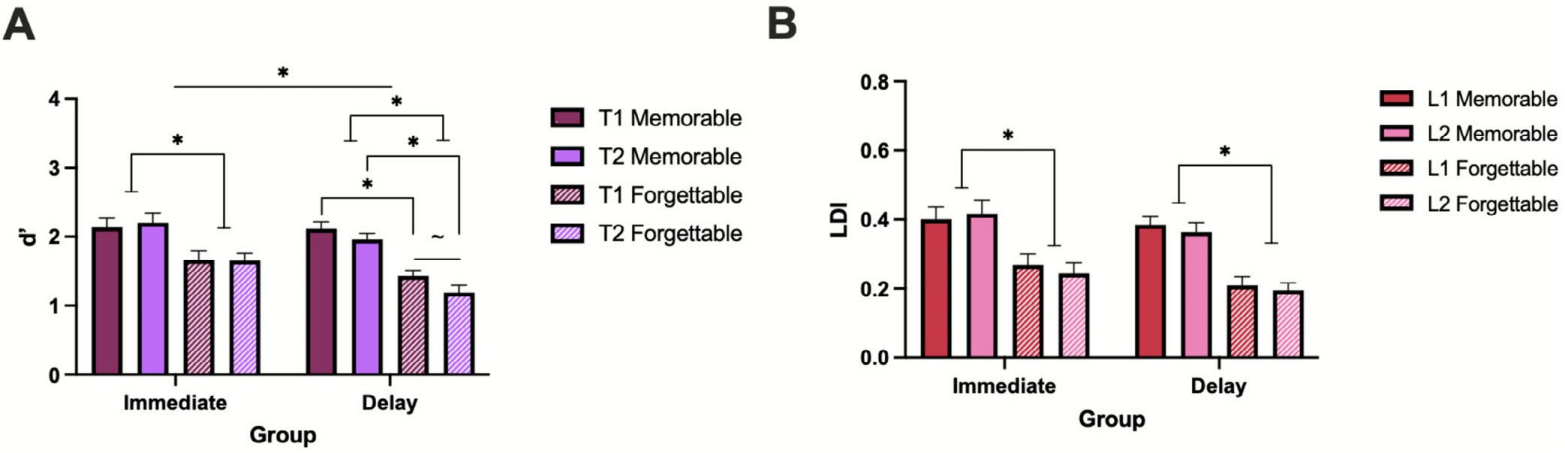
Memorability and presentation order effects on target recognition and lure discrimination. (A) Target recognition (*d*′) at immediate and delay testing times for memorable target first (T1 memorable), memorable target second (T2 memorable), forgettable target first (T1 forgettable), and forgettable target second (T2 forgettable). (B) Lure discrimination index (LDI) at immediate and delay testing times for memorable lure first (L1 memorable), memorable lure second (L2 memorable), forgettable lure first (L1 forgettable), and forgettable target second (L2 forgettable). Error bars represent SEM. *p ≤ 0.05. Immediate *N* = 25, delay *N* = 25.

Next, we set out to examine whether there were differences in our main memory measures of interest (*d*′ and LDI) that depended on the order in which the image pairs were presented to participants (lure first or second, and target first or second) in the modified task, as well as how these interacted with our variables of memorability, similarity, and time of testing. To examine the effects of presentation order on target recognition, we conducted a mixed repeated‐measures ANOVA with memorability (memorable, forgettable) and presentation order (T1, T2—i.e., target first or second) as within‐subjects factors, and group (immediate, delay) as the between‐subjects factor. We found a significant main effect of memorability [*F*(1,48) = 56.03, *p* < 0.001, $\eta_{p}^{2}=0.539$] and group [*F*(1,48) = 4.35, *p* = 0.042] (memorable > forgettable; immediate > delay). There was no significant main effect of presentation order [*F*(1,48) = 2.12, *p* = 0.15, $\eta_{p}^{2}=0.04$]. There was a marginally significant interaction between presentation order and group [*F*(1,48) = 3.86, *p* = 0.055, $\eta_{p}^{2}=0.07$], where there was worse memory for a target presented second (T2) relative to a target presented first (T1), but only after a delay, not immediately [*t* = 2.75, *p* = 0.046] (see Figure 6A). There was no interaction between presentation order and memorability (*p* = 0.35) and no three‐way interaction between presentation order, memorability, and group (*p* = 0.95).

We then examined presentation order and its effects on lure discrimination through a mixed repeated‐measures ANOVA with presentation order (L1, L2—i.e., lure first or second) and memorability (memorable, forgettable) as the within‐subjects factors and group (immediate, delay) as the between‐subjects factor. We found a main effect of memorability [*F*(1,48) = 83.77, *p* < 0.001, $\eta_{p}^{2}=0.636$], with better lure discrimination for memorable relative to forgettable images (Figure 6B). There was no significant main effect of presentation order (*p* = 0.42), nor of group (*p* = 0.19). The interactions between order and group (*p* = 0.59); memorability and group (*p* = 0.57); order and memorability (*p* = 0.54); and order, memorability, and group (*p* = 0.38) were not significant. When including similarity as an additional within‐subjects factor, there was no evidence of a significant impact of presentation order on LDI performance: order (*p* = 0.62); order and group (*p* = 0.52); order and memorability (*p* = 0.71); order, memorability, and group (*p* = 0.43); order and similarity (*p* = 0.07); order, similarity, and group (*p* = 0.07); order, memorability, and similarity (*p* = 0.77); four‐way (*p* = 0.17).

### 3.4 Correct Response Toward First Stimulus Enhanced Subsequent Memory for Second Stimulus

To explore differences in accuracy (correct, incorrect) identification of second targets and lures (T2, L2) based on the accuracy of their preceding paired conditions (L1, T1, respectively), we first examined the proportion of hits (accurately identifying a T2 image as “old”) or misses of a target presented second (incorrectly identifying a T2 image as “new”), given the CR (accurately identifying an L1 image as “new”) or false alarm (FA) (incorrectly identifying the L1 image as “old”) of its paired L1; as well as the inverse case for T1–L2. Because of the stimuli sample size, we were not powered to analyze paired memory accuracy across memorability or similarity. We analyzed a total of 50 cases for T1 and 48 for L1. The two exclusions occurred due to the randomization algorithm used for stimulus presentation in the experimental setup, where one of the conditions was not included for two participants.

**FIGURE 7:**
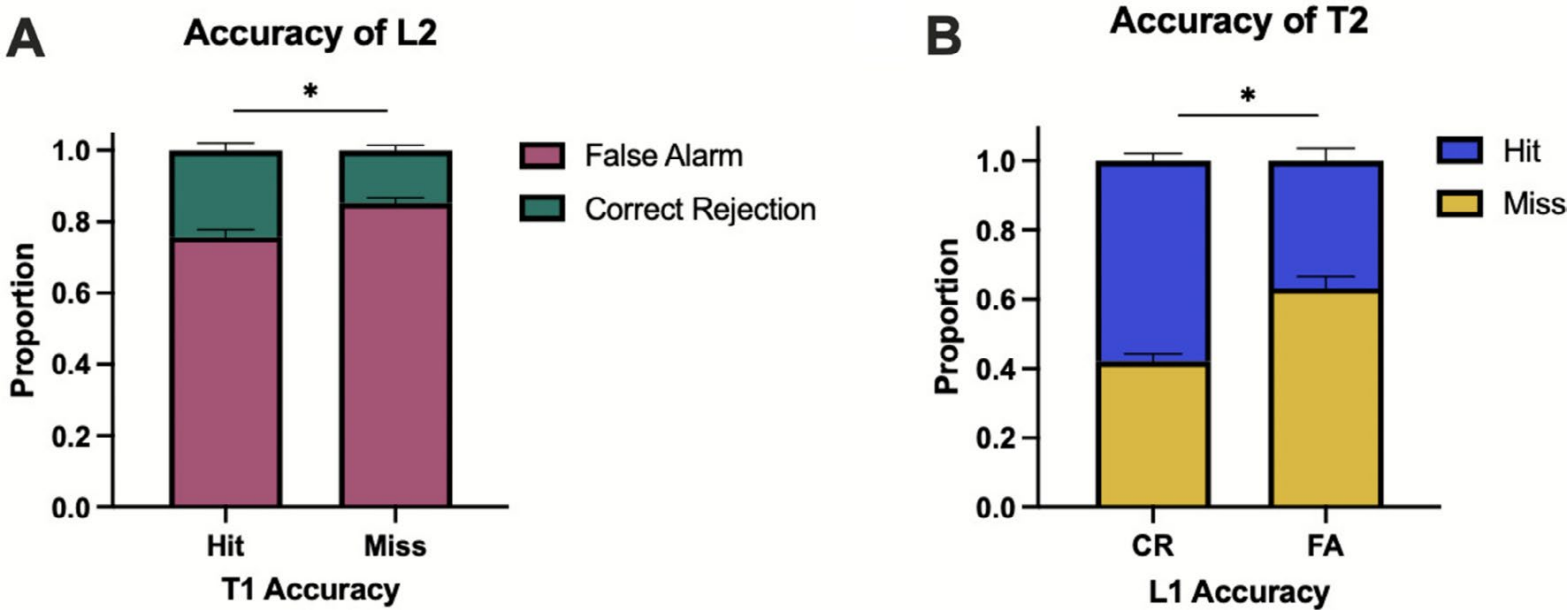
Influence of paired target–lure stimuli during retrieval on memory accuracy. (A) Differences in the proportion of correct rejections and false alarms across Hits and Misses of T1. (B) Differences in the proportion of hits and misses across CRs and FAs of L1s. Error bars represent SEM. *p ≤ 0.05. T1 Hit *N* = 50, T1 Miss *N* = 50, L1 CR *N* = 50, L1 FA *N* = 48.

Through paired samples *t*‐tests, we analyzed the difference in the proportion of CRs and FAs of L2s across Hits and Misses of T1s. Results showed a statistically significant difference [*t*(49) = 4.82, *p* < 0.001, *d* = 0.68], where correctly identifying a target first (T1) was associated with a higher proportion of subsequent CRs for its paired lure shown second (L2), whereas incorrectly calling a target first “new” (T1) was associated with a higher proportion of subsequent FAs for its paired lure shown second (L2) (Figure 7A).

When examining the hit accuracy of T2 based on L1 CRs and FAs, we found a statistically significant difference [*t*(47) = 5.26, *p* < 0.001, *d* = 0.76], where correctly rejecting a lure first (L1) yielded a higher proportion of Hits for its paired target shown second (T2). The inverse was also true, where incorrectly identifying a lure as “new” first (FA) was more likely to elicit a subsequent miss of its paired target presented second (T2) than a hit (Figure 7B).

## 4 Discussion

Understanding what factors influence how we remember has been a longstanding focus in memory research. While repetition has been shown to strengthen memory representations (Tulving and Schacter 1990), conflicting results have emerged regarding its role in facilitating or impairing memory. Factors such as interference (Yassa and Reagh 2013), attention (Kim et al. 2012), and stimulus properties such as similarity further complicate this relationship. Interference plays a critical role in memory processes, where prior memories may hinder the retention of new information, or where new memories disrupt the retrieval of older information (Yassa and Reagh 2013). These effects are influenced by the degree of overlap between competing memories, as well as the temporal spacing of learning episodes (Kim et al. 2012; Leal, Tighe, and Yassa 2014). High degrees of overlap or similarity in information often exacerbate interference, making it harder for the brain to effectively segregate and retrieve distinct memory traces (Yassa and Stark 2011). Additionally, memory resilience has been tied to memorability, with some studies demonstrating that memorable stimuli show greater resistance to forgetting across time delays (Oliva and Isola 2012). However, questions remain regarding how repetition and memorability interact with interference and whether these factors enhance or impair memory performance. In this study, we modified a MDT by including both repeated *and* lure images during retrieval and examined factors that may influence these effects such as memorability, similarity of lures, and time of testing. This experimental design provides a potential framework to assess how these factors impact the multifaceted relationship between recognition, discrimination, and the role of interference on these measures.

We found that target recognition was unaffected by the inclusion of repeated lure and target stimuli, while lure discrimination showed an interaction between this task modification and memorability, with exaggerated effects of memorability under conditions of greater interference during retrieval. While these effects are consistent with the possibility that interference may exaggerate memorability differences, they can also be understood within the broader MDT literature, which has demonstrated that performance on prior trials can result in biased discrimination of lures due to competition between encoding and retrieval (S. M. Stark et al. 2019). However, similar interference patterns have been observed in paradigms beyond MDTs, like those involving repetition and overlapping learning episodes (Reagh et al. 2017; Xue et al. 2010). The modified task used here may therefore engage interference processes from both the MDT and Competitive Trace Theory frameworks, reflecting a dynamic interplay between overlap, repetition, and retrieval monitoring rather than a single, isolated mechanism for interference.

Regarding the memorability literature, our findings align with previous work (Gillies et al. 2023) showing that memorable stimuli are more efficiently processed, though our data extend this by showing that retrieval interference further exaggerates these differences. This suggests that interference does not uniformly impact memory. We also found that the order of repeated target and lure stimuli during retrieval marginally impacted how we remember these experiences, such that repeated images presented first were better recognized than if they were presented second. However, this effect was marginal and was only evident after a 24‐h delay, suggesting time may exacerbate these order effects. Finally, we found that images that were presented first and remembered correctly influenced how images presented second were remembered, such that they were better remembered or discriminated. These findings suggest that recognition strength and mnemonic discrimination rely on distinct cognitive processes: repetition enhanced overall recognition performance (as indexed by *d*′), while at the same time inducing interference through overlapping exposures impaired the ability to discriminate lures from targets. However, it is also possible that memory for the second image may have been influenced not only by interference from the preceding image but also by the re‐encoding of both the stimulus and the participant's response to the first trial.

### 4.1 Interference Exaggerates Memorability Effects

Our results suggest that interference caused by repeated and overlapping exposures during retrieval interacts with memorability, with memorable images showing greater resilience and, in fact, better lure discrimination performance under conditions of greater interference compared to both memorable images under less interfering conditions as well as forgettable images. This is consistent with prior work demonstrating that memorable images are inherently more robust to forgetting (Gillies et al. 2023; Oliva and Isola 2012); however, our findings underscore how interference during retrieval can differentially boost or impair recall, pointing to a retrieval‐level contribution beyond an encoding‐focused account.

While we conceptualized the modified task as inducing greater interference, we acknowledge that the additional exposure to both target and lure images may also engage re‐encoding processes, where participants could have consolidated or updated memory traces based on their behavioral responses. Such response‐contingent encoding has been shown to modulate recognition (Kim et al. 2012) and may partially explain our results, where accuracy on the first stimulus influenced subsequent performance on the second. Thus, our design likely captures both interference and response‐dependent re‐encoding, which together contribute to the observed memory dynamics.

Our results may also reflect more general interference dynamics described in the mnemonic discrimination literature. MDT work has shown that competition between overlapping memory traces, particularly between targets and lures, can bias subsequent retrieval decisions and modulate discrimination performance (Reagh and Yassa 2014b; S. M. Stark et al. 2019). Our findings could be reflecting a broader process that amplifies preexisting differences in memory strength. Our current findings provide strong evidence of interference driven by target–lure similarity, a hallmark of the MDT (Kirwan and Stark 2007; C. E. L. Stark et al. 2023). This effect was robust across both experiments and consistent across memorability and time delays. While our modified test format was designed to increase retrieval interference, it may not induce a novel interference mechanism but instead modulate existing interference dynamics through multiple overlapping exposures that heighten retrieval competition.

While previous research has emphasized the negative effects of interference on episodic memory (Kim et al. 2012; Reagh and Yassa 2014b), our findings complicate this narrative and suggest that repeated exposure to similar (but nonidentical) stimuli during retrieval may serve to reinforce distinctive features of memorable items, perhaps by engaging hippocampal pattern separation more effectively or by strengthening associative pathways. This interpretation is compatible with frameworks which posit that multiple overlapping traces can strengthen or distort memory depending on context (e.g., if they are sufficiently distinct) (Nadel et al. 2000; Yassa and Reagh 2013). Under this view, our design's repeated and overlapping presentations may have reactivated related memory traces, leading to both memory enhancement and interference. Interference may act as a form of “retrieval practice” or elaborative encoding when the memory is strong enough to support it. These effects parallel findings from studies of retrieval‐based learning, where repeated but variable retrieval efforts strengthen memory (Roediger and Butler 2011).

For target recognition, memorable images outperformed forgettable ones across both immediate and delayed retrieval, and this effect persisted under conditions of increased interference in our modified task. Notably, memory for memorable images was resilient even after a 24‐h delay, suggesting that repetition and interference interact to strengthen robust memory traces while simultaneously amplifying differences between memorable and forgettable images. Specifically, memorable images showed better target recognition and lure discrimination compared to forgettable images across both immediate and delayed retrieval, where the effect of memorability became even more pronounced under increased interference conditions. For lure discrimination, we observed the possibility that interference may differentially impact the discrimination performance of forgettable images. However, other factors, including retrieval demands, could have also contributed to the observed effects. Where the observed asymmetry could suggest that memorable items may benefit from enhanced encoding or reactivation during repeated retrieval, while forgettable items are more susceptible to interference effects in the modified task. These findings could contribute to the broader literature by highlighting the complex relationship between interference and memorability. While previous studies have shown that repetition can impair lure discrimination (Reagh and Yassa 2014a), our results suggest that this impairment is not uniform. Instead, the degree of interference is modulated by memorability, with memorable items showing greater resistance to interference and forgettable items showing greater susceptibility. This interaction emphasizes that memorability plays a critical role in determining the resilience of memory representations, even under conditions of increased interference.

### 4.2 The Role of Similarity in Repetition and Interference

Our results underscore the critical role of similarity in shaping what we remember. In our study, when stimuli between encoding and retrieval were highly similar, lure discrimination was hampered. This may be due to the potential creation of overlapping memory traces, making it difficult to remember specific images. This finding supports prior work showing that multiple exposures of an event (either identical or similar) can reduce attention to subtle differences between similar events (Kim et al. 2012; Reagh and Yassa 2014a). In contrast, when similarity between encoding and retrieval was low, we found better lure discrimination, as fewer overlapping traces may be competing for representation. These results demonstrate that stimulus similarity can modulate the balance between the facilitating and interfering effects of repetition. These simultaneous findings show that repetition may strengthen recognition memory while also hindering discrimination in detailed memory.

### 4.3 The Role of Presentation Order and Accuracy

Contrary to our hypotheses, the order in which targets and lures were presented during retrieval (e.g., target first vs. target second) did not significantly affect overall memory performance. This finding was somewhat unexpected, as we predicted that second presentations (e.g., T2 or L2) would suffer from interference due to the preceding presentation of a similar image. However, a marginal effect emerged such that delayed retrieval of second targets (T2) showed slightly worse recognition compared to immediate retrieval. This could suggest that the impact of presentation order may depend on the retention interval and possibly the decay or consolidation of the first‐presented image. Larger sample sizes will be required to make any strong inferences about presentation order and interactions with time.

Interestingly, while overall presentation order did not significantly influence mnemonic discrimination or target recognition, our exploratory analyses revealed that the accuracy of first responses (e.g., correctly rejecting a lure first) had a meaningful influence on subsequent paired responses (e.g., correctly identifying a target second). Specifically, correctly rejecting the lure presented first (L1) enhanced recognition of the subsequent paired target (T2), and conversely, FAs to the first image led to more errors on the second. These findings highlight a dynamic interaction between paired memory judgments, potentially reflecting feedback or updating processes that unfold during retrieval. These results also indicate that participants may have relied on memory for both the first presentation and their response to it when evaluating the second paired trial. Because no feedback was provided, this would reflect internally generated monitoring processes rather than feedback‐based learning. Such response‐monitoring processes are consistent with frameworks of retrieval‐based updating, in which reactivation of overlapping traces, including one's own prior response, can influence subsequent retrieval decisions (Polyn et al. 2009; Yassa and Reagh 2013). Consistent with this interpretation, additional analyses comparing the first presentations (T1s and L1s) across the two task formats showed no overall differences in accuracy, suggesting that the modified task did not globally impair recognition or discrimination. However, interactions between memorability and task emerged for L1s, with forgettable and low‐similarity items showing the largest decrements, consistent with the idea that the modified format exaggerates memorability‐related effects rather than introducing a uniform performance change (see Supporting Information 7 in Data S1). Thus, the modified experimental design likely leads to multiple facets of interference (e.g., learning there are two images related to the baseline image, memory of response on first trial in Experiment 2, which image is shown first). Future studies should explore accuracy patterns more systematically by increasing stimulus samples, providing feedback to measure response monitoring, and examining how order effects evolve with repeated exposures, as well as investigating potential mechanisms, such as neural correlates of interference resolution.

### 4.4 Neurobiological Mechanisms Underlying Memorability and Interference

Understanding the neural mechanisms underlying memorability could provide insight into the interactions between perception and memory. Gaining a clearer role of the MTL and hippocampal subregions in driving the memorability of a stimulus will be important to determine given each region's unique role in memory processing. Previous high‐resolution imaging studies examining neurobiological mechanisms during lure discrimination have observed engagement of the hippocampal DG and CA3 as well as the perirhinal cortex (Kirwan and Stark 2007; Lacy et al. 2011; Leal, Tighe, Jones, and Yassa 2014; Reagh et al. 2017; Stevenson et al. 2020). It has been suggested that hippocampal pattern separation could be a candidate mechanism underlying memorability (Kramer et al. 2023). This is supported by the finding that the brain is sensitive to image memorability, regardless of memory performance (Bainbridge and Rissman 2018), where there is a representational organization in the brain based on memorability; with memorable items showing a tendency to be highly similar, while forgettable images are more dissimilar from one another (Bainbridge et al. 2017). This is akin to work showing higher representational similarity in pattern completion and higher distinctiveness in pattern separation (Larocque et al. 2013), pointing toward a potential connection between the two.

Our studies were behavioral in nature; thus, we cannot make direct claims about the underlying neural mechanisms driving our findings. Future studies using high‐resolution neuroimaging techniques of the hippocampal regions, alongside regions of perception, will be important toward elucidating the neural mechanisms underlying the interactions between memorability and encoding/retrieval memory processes and the processes that enable memorable images to resist interference.

Based on previous studies, we hypothesize greater DG/CA3 activity during the CR of memorable lure stimuli compared to forgettable stimuli. Under conditions of increased interference, we expect these differences to be exaggerated, especially in the DG/CA3 compared to other hippocampal subfields (e.g., CA1) and target recognition measures (repeated images). Alternatively, activity during lure CRs may be independent of memorability representation in the brain, where ventral visual areas and memory‐related areas have been shown to be associated with stimulus memorability but independent of memory performance, pointing toward a role in supporting visual recognition memory behavior during memory encoding. Studying hippocampal subfields and connectivity with perceptual regions throughout the brain using high‐resolution imaging will allow us to determine how memorability and interference influence this network.

### 4.5 Limitations, Future Directions, and Conclusions

Based on three theoretical frameworks, our study aimed to address longstanding debates in the literature regarding the effects of repetition and interference on memory performance. By incorporating measures of both target recognition (*d*′) and lure discrimination (LDI), as well as our modified version of a standard MDT, we provide a more nuanced understanding of how repetition and interference interact with similarity and memorability to influence memory performance. The dissociation between *d*′ and LDI on the interaction between memorability and interference underscores the importance of using sensitive memory measures and tasks to capture subtle effects. Additionally, our findings help bridge the gap between the memorability and pattern separation literatures, demonstrating that memorable images exhibit enhanced resilience to interference, even under conditions that typically impair lure discrimination (e.g., greater interference). The observation that interference exaggerates the effects of memorability suggests that memory resilience is not merely a function of time delays but is also influenced by the degree of interference during retrieval.

While our findings provide important insights, there are important limitations to consider and future directions to further our understanding of these complex interacting factors in memory. One limitation of our task design is the categorization of images into binary groups of memorable and forgettable, as this may oversimplify what is inherently a continuous construct. This can obscure more nuanced, graded effects of memorability on memory performance and reduce sensitivity to subtle variability in stimulus properties. We adopted a threshold of 0.80, consistent with prior memorability research to maximize statistical power by including all images in our MDT (Rust and Mehrpour 2020). This approach avoided excluding images near the threshold and instead allowed for comparisons across a broader range of memorability conditions. Future work may benefit from treating memorability as a continuous variable to better capture the spectrum of image‐level differences and their interaction with individual and contextual factors. Additionally, lure similarity was based on ratings from an independent sample using a numerical scale. Future studies could refine how similarity is defined and perceived by participants (e.g., using machine‐learning or image detection algorithms) to better understand the factors driving our findings.

Another methodological limitation is our between‐subjects design, where the immediate and delay groups were independent of each other. This prevented direct within‐subject comparisons of memory performance immediately and after 24 h. Future research should explore within‐subject designs to examine how memorability and memory performance evolve over time. In addition, our sample sizes were relatively small, which makes us less powered to detect small effects. Future larger scale studies would be beneficial to better characterize individual differences that may contribute to these findings. Regarding sample characteristics, our findings may not fully generalize due to the demographic constraints of our participant pool. We recruited cognitively healthy young adults (ages 18–35) to establish baseline behavior for this experimental paradigm. Future studies should include a wider age range and varying educational backgrounds to determine whether these effects persist across different populations, as recent studies have shown population‐level differences in memorability effects across cultures (Chen et al. 2025). Examining differences in memorability‐based mnemonic discrimination across groups may provide insights into memory deficits in aging and age‐related cognitive decline, as well as the associated neural mechanisms.

Furthermore, the memorability scores in our study were derived from an existing dataset where behavioral memorability scores were collected from a European population (Goetschalckx and Wagemans 2019). Our sample, collected in the United States, had a more racially and ethnically diverse composition. While memorability is generally considered consistent across observers, it is possible that some of the observed effects stem from cultural differences, particularly when stimuli (e.g., food, landscapes) are less familiar to one group than another. Furthermore, we were unable to systematically examine interactions between memorability and accuracy‐related measures due to too few trials to correctly characterize this. While we assessed how the accuracy of an initial stimulus (T1 or L1) influenced subsequent recognition (T2 or L2), we did not explore whether an image's inherent memorability modulated this effect. For instance, highly memorable stimuli may be more resistant to interference, while less memorable ones may be more susceptible. Future research could investigate whether memorability interacts with retrieval accuracy to determine whether the observed effects are specific to certain types of stimuli. Additionally, examining the effects of multiple forms of interference (e.g., repetitions and lures) during retrieval could clarify whether the observed facilitation and interference effects are maintained or amplified with increased exposure. Moreover, a limitation of our paradigm is that the modified task may not isolate interference, as we have discussed above; additional exposures could promote re‐encoding or response‐based learning, where improved accuracy for second items could partly reflect participants' internal monitoring of their prior response, even though no feedback was provided. Future studies could disentangle these effects by including conditions that vary the opportunity for re‐encoding or by incorporating confidence measures to track retrieval monitoring. Finally, as mentioned above, our findings are limited by being purely behavioral in nature. By leveraging both behavioral and neural measures, future research can build on the current findings to develop a more comprehensive understanding of how repeated experiences shape memory resilience and discrimination.

### 4.6 Conclusions

This study demonstrates that memorability is not only predictive of recognition accuracy but also modulates the effects of interference caused by repetition and overlap during retrieval on lure discrimination in a MDT. Memorable images exhibited resilience and even benefits from increased interference, while forgettable images suffered under the same conditions. These findings suggest that memorability may interact with pattern separation processes in the hippocampus to support memory precision and highlight the importance of considering intrinsic stimulus features when evaluating memory performance.

## Author Contributions

**Fernanda Morales‐Calva:** conceptualization, data collection, formal analysis, data curation, investigation, writing – original draft, writing – review and editing, visualization, supervision. **Aditi Velgekar:** data collection, writing – original draft, visualization. **Stephanie L. Leal:** formal analysis, writing – review and editing, and supervision.

## Funding

F.M.C. was supported by a Fulbright‐COMEXUS García‐Robles grant. This work was supported by a Rice University School of Social Sciences Research Institute Pre‐Dissertation Research Grant to F.M.C.; A.V. was partially supported with funds from Rice University's Department of Psychological Sciences Gertrude Maurin Fund; S.L.L. is supported by a BrightFocus Foundation Alzheimer's Association Research Grant (A2022040S), a NARSAD Brain & Behavior Research Foundation Young Investigator Grant (#30897), and an Alzheimer's Association Grant (24HPE‐1287002).

## Conflicts of Interest

The authors declare no conflicts of interest.

## Supporting information


**Data S1:** Supporting information.

## Data Availability

The data that support the findings of this study are openly available in GitHub at https://github.com/lealmemorylab/interference_memorability.
