# Supplementary material for: Greater Interference From Multiple Exposures During Memory Retrieval Drives More Memorable and Forgettable Experiences
Source: Hippocampus. 2025 Dec 23;36(1):e70057. doi: 10.1002/hipo.70057 (PMC12728388; doi:10.1002/hipo.70057)
Supplement: Supplementary file 1 — Data S1: Supporting information. [file HIPO-36-0-s001.docx]

**Data S1: Supplementary Materials**

**Supporting Information 1: Questionnaires**

Participants were administered a series of questionnaires related to lifestyle factors. They completed 1) a Medical Screening Questionnaire to collect information related to their medical history. They also completed 2) a demographics form and 3) a modified version of the PSQI. Participants in the 24-hour delay condition completed 4) a second version of the modified PSQI asking about their sleep the previous night. All research participants completed 5) the Perceived Stress Scale (S. Cohen, 1988; S. Cohen et al., 1983), which assesses individual stress levels currently and during the previous month. The 6) BDI and 7) BAI to measure depression and anxiety, respectively. Participants who indicated a positive response to the question related to suicidal ideation in the BDI, were additionally given 8) a SISQ and community resources after participation. Female and non-binary participants were given 9) a Birth Control Questionnaire (BCQ) asking about their current use of hormonal contraceptives. All participants also completed 10) a Lifestyle and Exercise Questionnaire (LEQ) which asks about levels of physical activity and dietary habits. 11) Participants were assessed on their alcohol consumption habits through the Alcohol Screening Questionnaire (AUDIT) (Johnson et al., 2013). Finally, they were asked to complete 12) the ATTC, and 13) learning styles questionnaires.

**Supporting Information 2**

Table S2-1. Stimuli shown during the encoding phase of the Modified MDT

| Encoding | | | |
| --- | --- | --- | --- |
|  | Memorable | Forgettable | Total |
| Baseline (not repeated) | 40 | 40 | 80 |
| Animals | 8 | 8 | 16 |
| Food | 13 | 6 | 19 |
| Landscape | 4 | 9 | 13 |
| Sports | 11 | 11 | 22 |
| Vehicle | 4 | 6 | 10 |
| High Sim Baseline Lure | 21 | 19 | 40 |
| Animals | 5 | 2 | 7 |
| Food | 5 | 1 | 6 |
| Landscape | 2 | 6 | 8 |
| Sports | 5 | 7 | 12 |
| Vehicle | 4 | 3 | 7 |
| Low Sim Baseline Lure | 20 | 20 | 40 |
| Animals | 3 | 0 | 3 |
| Food | 6 | 0 | 6 |
| Landscape | 0 | 10 | 10 |
| Sports | 5 | 4 | 9 |
| Vehicle | 6 | 6 | 12 |
| TOTAL | 81 | 79 | 160 |

Table S2-2. Stimuli shown during the retrieval phase of the Modified MDT

| Retrieval | | | |
| --- | --- | --- | --- |
|  | Memorable | Forgettable | Total |
| High Sim Baseline (Target) | 21 | 19 | 40 |
| Animals | 5 | 2 | 7 |
| Food | 5 | 1 | 6 |
| Landscape | 2 | 6 | 8 |
| Sports | 5 | 7 | 12 |
| Vehicle | 4 | 3 | 7 |
| Low Sim Baseline (Target) | 20 | 20 | 40 |
| Animals | 3 | - | 3 |
| Food | 6 | - | 6 |
| Landscape | - | 10 | 10 |
| Sports | 5 | 4 | 9 |
| Vehicle | 6 | 6 | 12 |
| High Sim Lure | 21 | 19 | 40 |
| Animals | 5 | 2 | 7 |
| Food | 5 | 1 | 6 |
| Landscape | 2 | 6 | 8 |
| Sports | 5 | 7 | 12 |
| Vehicle | 4 | 3 | 7 |
| Low Sim Lure | 20 | 20 | 40 |
| Animals | 3 | 0 | 3 |
| Food | 6 | 0 | 6 |
| Landscape | 0 | 10 | 10 |
| Sports | 5 | 4 | 9 |
| Vehicle | 6 | 6 | 12 |
| Foil | 39 | 41 | 80 |
| Animals | 10 | 8 | 18 |
| Food | 8 | 8 | 16 |
| Landscape | 6 | 10 | 16 |
| Sports | 8 | 8 | 16 |
| Vehicle | 7 | 7 | 14 |
| Total | 121 | 119 | 240 |

**Supporting Information 3**


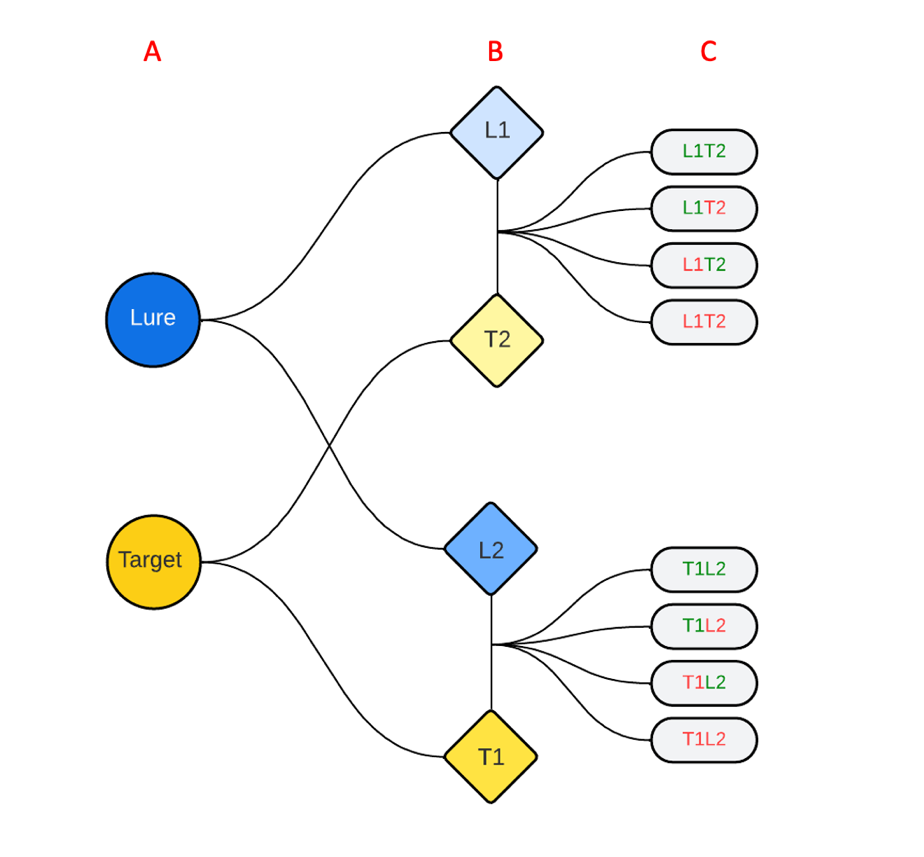


Figure S3. Data analysis levels for the interference version of the memorability-based mnemonic discrimination task. Analyses for d’ and LDI were performed at the (A) individual lure (blue circle) and target (yellow circle) level. (B) Analyses were also performed comparing presentation order (yellow squares, T1 vs T2) and (blue squares, L1 vs L2).

**Supporting Information 4**

Table S3. *Summary of Proportion Correct Responses on Standard and Modified Task*

| Measures | STANDARD TASK | | | MODIFIED TASK | | |
| --- | --- | --- | --- | --- | --- | --- |
|  | Immediate | Delay | Immediate | | Delay | |
| Memorable Target Hits | 0.66 ± .15 | 0.53 ± .14 | | 0.57 ± .18 | 0.56 ± .11 | |
| Memorable Foil CR | 0.95 ± .10 | 0.94 ± .05 | | 0.97 ± .03 | 0.96 ± .03 | |
| Memorable Lure CR | 0.79 ± .13 | 0.75 ±.14 | | 0.83 ± .08 | 0.80 ± .12 | |
| Forgettable Target Hits | 0.58 ± .15 | 0.45 ± .14 | | 0.41 ± .16 | 0.34 ± .12 | |
| Forgettable Foil CR | 0.92 ± .10 | 0.92 ± .05 | | 0.96 ± .04 | 0.95 ± .03 | |
| Forgettable Lure CR | 0.81 ± .12 | 0.78 ± .11 | | 0.83 ± .13 | 0.85 ± .09 | |
| High Similarity Lure CR | 0.74 ± .13 | 0.71 ± .12 | | 0.78 ± .11 | 0.78 ± .12 | |
| Low Similarity Lure CR | 0.87 ± .11 | 0.82 ± .11 | | 0.88 ± .09 | 0.87 ± .09 | |
|  |  |  | |  | |  |

Note: CR: Correct Rejections. All measurements represent Mean ± Standard Deviation

**Supporting Information 5**

We aimed to analyze the interaction between memorability and time of testing and their potential differences across experimental settings (standard vs. modified). For target recognition, we conducted a repeated-measures ANOVA with memorability (memorable, forgettable) as the within-subjects factor and task (standard, modified) and time of testing (immediate, delay) as a between-subjects factor. There was a main effect of memorability, [*F(1,98) =* 88.5, *p* < .001, η_p_^2^ = .47], in which memorable images were better remembered than forgettable images [Figure 5A]. As expected, there was a significant main effect of time [*F(1,98) =* 11.9, *p* < .001, η_p_^2^ = .109], where those tested immediately showed better target recognition than those tested after 24hrs. There were no main effects of task, nor significant interactions between memorability and task; memorability and time; memorability, time and task; or task and time (all *p’*s > .05).


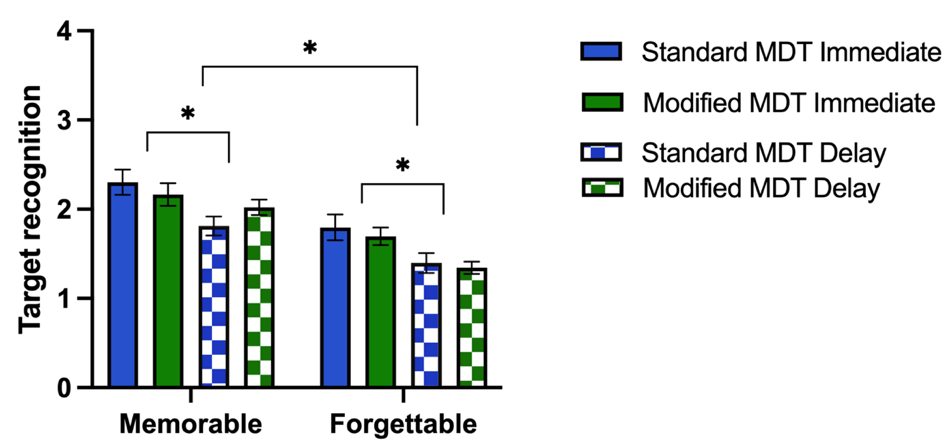


**Figure S5. The impact of increased interference on memorable and forgettable images in target recognition across time delays**. Target recognition (d’) performance for memorable and forgettable images on standard (blue) and modified (green) mnemonic discrimination tasks (MDT) across immediate (solid) and delay (checkered) times of testing. Error bars represent SEM. * = p ≤ .05.

**Supporting Information 6**

For lure discrimination, we conducted a repeated-measures ANOVA with memorability (memorable, forgettable) and lure similarity (high, medium, low) as within-subjects factors and task (standard, modified) and time of testing (immediate, delay) as between-subjects factors. There was a main effect of memorability [*F(1,98) =* 91.5, *p* < .001, η_p_^2^ = .48], with memorable images being better discriminated than forgettable ones. There was a main effect of similarity [*F(2,196) =* 83.18, *p* < .001, η_p_^2^ = .46], where higher levels of lure discrimination were evident as lure similarity decreased. There was a significant interaction between similarity and memorability [*F(2,196) =* 9.89, *p* < .001, η_p_^2^ = .092], where memorable items showed better lure discrimination as lure similarity decreased [See Fig 5B]. There was a significant interaction between memorability and task [*F(1,98) =* 33.66, *p* < .001, η_p_^2^ = .26]. Interestingly, memorable images from the modified task were better remembered compared to standard task performance, and the opposite was true for forgettable images, where the modified task showed worse lure discrimination for forgettable images compared to standard task performance. Additionally, we found a significant main effect of testing time [*F(1,98) =* 13.8, *p* < .001, η_p_^2^ = .123], with participants tested immediately outperforming those tested after 24 hours. We also found a significant interaction between task and time [*F(1,98) =* 4.48, *p* = .037, η_p_^2^ = .044], with larger differences shown between immediate and delay testing in the standard task than in the modified task. There were no main effects of task, nor significant interactions between memorability and time; memorability, time and task; similarity and time; similarity and task; similarity, time and task; memorability, similarity and time; memorability, similarity and task; nor a four-way interaction.


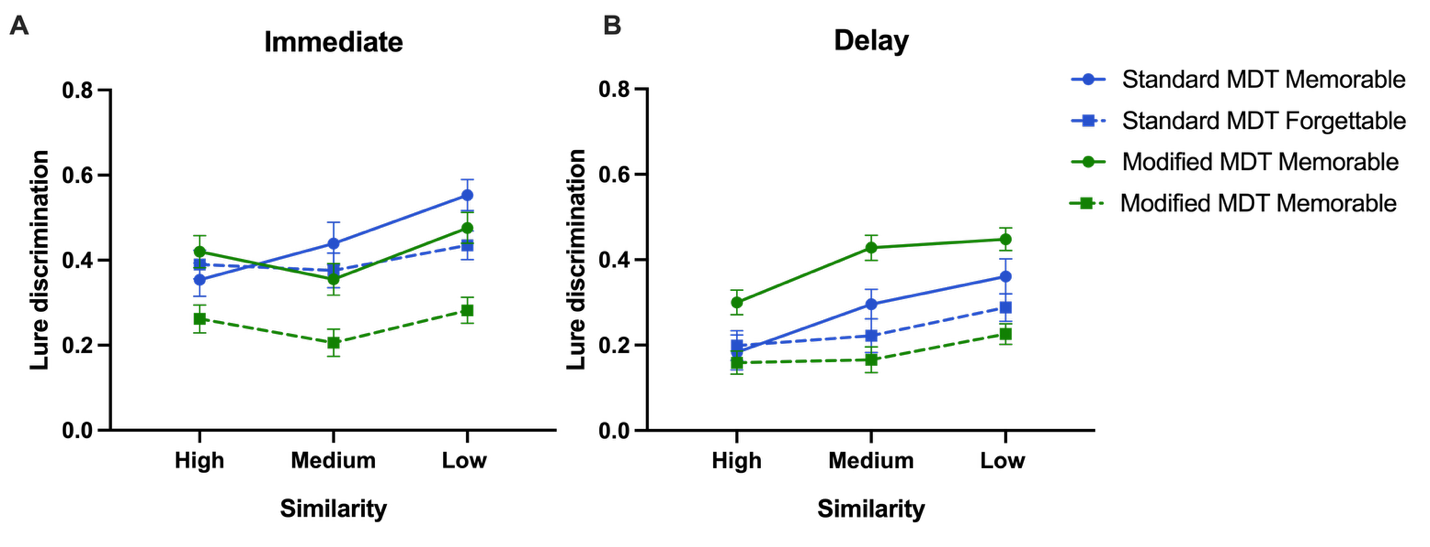


**Figure S6. The impact of increased interference from multiple exposures on memorable and forgettable images in Lure Discrimination across time delays.** Lure discrimination index (LDI) performance for memorable (continuous) and forgettable (dotted) images on standard (blue) and modified (green) tasks across immediate (A) and delay (B) testing times. MDTs. Error bars represent SEM.

**Supporting Information 7**

To examine how time of testing and task format influenced the effects of item memorability on target recognition performance across T1s in Experiment 2 and targets in Experiment 1, we ran a repeated measures ANOVA with time (immediate and delay) and task (standard and modified) as between-subjects factors and memorability (memorable and forgettable) as the repeated measures factor. As expected, we found a main effect of **memorability,** F(1, 98) = 79.93, p < .001, η_p_^2^ = .449, with memorable items outperforming forgettable ones in target recognition. The analysis also revealed the expected significant main effect of **time,** F(1, 98) = 7.24, p = .008, η_p_^2^= .069, indicating that participants tested immediately performed better than those tested after a delay. Importantly, the main effect of **task** was not significant, F(1, 98) = 0.01, p = .928, partial η_p_^2^ < .001, suggesting that overall performance on T1s and targets across Experiment 2 and 1, respectively, did not differ. The **time and task, memorability and task, memorability and time, or the three-way interactions were not statistically significant** (all *p’*s > .05)**.**

We ran a similar analysis for LDI, with time and task as between-subjects factors and with memorability and similarity (high and low) as repeated-measures factors for LDI performance in L1s for Experiment 2 and all lures in Experiment 1. Again, the main effects of memorability [F(1, 97) = 58.39, p < .001], time [F(1, 97) = 9.86, p = .002], and similarity [F*(1, 97)* = 38.35, p < .001] were significant. The main effect of task was not statistically significant, F*(1, 97)* = 1.09, p = .299, suggesting that overall performance did not differ for L1s in Experiment 2 and lures in Experiment 1. However, there were significant interactions between memorability and task F(1, 97) = 14.00, p < .001, where forgettable items were worse discriminated in the modified task compared to the standard one, suggesting that the effect of memorability differed across task formats. Additionally, the similarity and task interaction was significant, F(1, 97) = 7.60, p = .007, with lower similarity items suffering more from incorrect discrimination in L1 presentations in the modified task compared to general LDI on the standard task. The time and task interaction was also significant, F(*1, 97)* = 5.76, p = .018, where there were differences across time of testing in the standard version but no differences in the modified version. Finally, there was a significant interaction between memorability and similarity, F*(1, 97)* = 9.10*, p* = .003, such that performance for high-similarity memorable items did not differ significantly from that of low-similarity forgettable items. This effect reflects that the advantage for memorable items diminished under high similarity, such that high-similarity memorable items performed similarly to low-similarity forgettable ones. All other interactions were not statistically significant (all *p’*s > .05).

The analyses revealed that overall performance for first presentations (T1s and L1s) in the modified task did not differ significantly from the corresponding targets and lures in the standard task, indicating that the modified format did not globally alter recognition or discrimination for initial exposures. For **T1s**, there were significant main effects of memorability and time*,* but no main effect or interactions involving task. This pattern suggests that, because T1s represent first presentations, the two task formats did not differ at this stage; rather, both involved initial encoding under comparable conditions, resulting in equivalent performance across tasks.

For L1s, there were significant main effects of memorability, similarity, and time, along with several interactions involving task. Although there was no main effect of task, forgettable and low-similarity items were disproportionately affected in the modified task, suggesting that LDI was more sensitive to task format when discrimination was more difficult. This pattern indicates that the observed differences were not driven solely by the task itself, but by how specific stimulus properties interact with the added demands of the modified version. Nonetheless, the significant interactions for lure items suggest that the modified task imposes additional cognitive load or interference, particularly under more challenging item conditions. Moreover, because first and second presentations were interspersed rather than blocked, participants continuously alternated between encoding and retrieval demands, making the modified task inherently more difficult. Thus, factors such as fatigue, increased attentional demands, or overlapping retrieval processes may contribute to these effects. Finally, the significant memorability and similarity interaction showed that discrimination performance for high-similarity memorable items was comparable to that for low-similarity forgettable item, suggesting that high similarity reduces the typical memorability advantage. Together, these findings show that for first presentations, target recognition was equivalent across task versions, whereas lure discrimination was more sensitive to task format, possibly reflecting the heightened cognitive demands and with the idea that the modified task engages retrieval-like or interference-prone processes even during initial exposures.
